# Characteristics of chronic enteropathy associated with *SLCO2A1* gene (CEAS) in children, a unique type of monogenic very early-onset inflammatory bowel disease

**DOI:** 10.1186/s12887-024-04877-x

**Published:** 2024-06-18

**Authors:** Jin Gyu Lim, Jae Sung Ko, Jung Min Ko, Hyun Young Kim, Man Jin Kim, Moon Woo Seong, Young Hun Choi, Gyeong Hoon Kang, Jaemoon Koh, Jin Soo Moon

**Affiliations:** 1 Division of Pediatric Gastroenterology, Hepatology and Nutrition, Department of Pediatrics, Seoul National University College of Medicine, Seoul National University Children’s Hospital, 101 Daehak-Ro, Jongno-Gu, Seoul, 03080 Korea; 2 Department of Pediatric Surgery, Seoul National University College of Medicine, Seoul, Korea; 3 Department of Laboratory Medicine, Seoul National University College of Medicine, Seoul, Korea; 4 Department of Radiology, Seoul National University College of Medicine, Seoul, Korea; 5 Department of Pathology, Seoul National University College of Medicine, Seoul, Korea

**Keywords:** Inflammatory bowel disease, SLCO2A1 gene, Human, Intestine, Small

## Abstract

**Background:**

Chronic enteropathy associated with *SLCO2A1* gene (CEAS) is a unique type of inflammatory bowel disease. CEAS is monogenic disease and is thought to develop from childhood, but studies on pediatric CEAS are scarce. We analyzed characteristics of pediatric CEAS.

**Methods:**

Eleven patients diagnosed with CEAS at Seoul National University Children’s Hospital were identified and analyzed. Clinical data of patients were collected. Sanger sequencing of *SLCO2A1* was performed on all patients.

**Results:**

Patients were diagnosed at a median age of 16.0 years (IQR 11.0 ~ 20.0), and the median age at symptoms onset was only 4.0 years (IQR 2.5 ~ 6.0). Growth delay was observed at the time of diagnosis. Patients showed multiple ulcers or strictures in the small intestine, while the esophagus and colon were unaffected in any patients. Almost half of the patients underwent small intestine resection. The major laboratory features of pediatric CEAS include iron deficiency anemia (IDA), hypoalbuminemia, and near-normal levels of C-reactive protein (CRP). Two novel mutations of *SLCO2A1* were identified. The most prevalent symptoms were abdominal pain and pale face. None of the immunomodulatory drugs showed a significant effect on CEAS.

**Conclusions:**

Pediatric CEAS typically develop from very young age, suggesting it as one type of monogenic very early onset inflammatory bowel disease. CEAS can cause growth delay in children but there is no effective treatment currently. We recommend screening for *SLCO2A1* mutations to pediatric patients with chronic IDA from a young age and small intestine ulcers without elevation of CRP levels.

## Background

The incidence of inflammatory bowel disease (IBD) in children is steadily increasing worldwide, and many studies are currently being conducted to find the genetic susceptibility of IBD. [1–4] Chronic enteropathy associated with *SLCO2A1* gene (CEAS) is a recently reported rare type of IBD caused by recessive mutations in *SLCO2A1*. In 1968, Okabe et al*.* first described a unique type of chronic enteropathy with multiple shallow ulcers in the small intestine, called chronic nonspecific multiple ulcers of the small intestine (CNSU). [5, 6] Cryptogenic multifocal ulcerous stenosing enteritis (CMUSE), which has similar clinical features with CNSU, has also been reported in Korea. [7] In 2015, Umeno et al*.* found recessive mutations of *SLCO2A1* in some CNSU patients, and the disease called CEAS was established. [8–10] Furthermore, *SLCO2A1* mutation is also known as the cause of primary hypertrophic osteoarthropathy (PHO) characterized by pachydermia, digital clubbing, and periostosis. [11, 12].

CEAS is known to cause multiple ulcers and stricture in the gastrointestinal (GI) tract. In addition, CEAS is considered to be underdiagnosed or misdiagnosed, because this disease is not well known and characteristics are similar to those of other GI diseases such as eosinophilic gastrointestinal disease (EGID) or Crohn’s disease. Until now, most reports on CEAS have been primarily from Asia, with only one case reported in Europe so far. [8, 10, 13–21].

Over 80 monogenic variants known to cause IBD have been identified to date, and they are referred to as monogenic IBD. It typically manifests from early childhood, making it a crucial factor in pediatric IBD. [22] CEAS is also a monogenic GI tract disease but in the 2020 position paper on very early onset inflammatory bowel disease (VEO-IBD) by the North American Society for Pediatric Gastroenterology, Hepatology and Nutrition, CEAS was not included. [23] Recently, based on a single case series from Japan, CEAS is gradually being recognized as a subtype of VEO-IBD. [24, 25] Nevertheless, the identified cases of pediatric CEAS are still limited, and the literature remains scarce.

In light of this, this study was aimed to identify pediatric CEAS in Seoul National University Children’s Hospital (SNUCH). Additionally, we conducted a detailed analysis of the characteristics and clinical response to treatment of pediatric CEAS to gain a clear perspective of the disease.

## Method

### Study participants

In 2018, Umeno *at el.* first proposed clinical criteria for CEAS. [9] Based on this, the diagnosis of CEAS was confirmed by combining chronic GI symptoms, evidence of intestinal lesions by endoscopy, and genetic confirmation of recessive *SLCO2A1* mutations.

This study is a retrospective single center study of pediatric CEAS. We conducted a genetic test of *SLCO2A1* in patients with chronic GI ulceration disease who did not exhibit typical features of major IBD such as ulcerative colitis or Crohn’s disease, or had been diagnosed with CMUSE previously at SNUCH. Through this, out of a total of 28 patients screened, and eleven patients diagnosed with CEAS were identified and enrolled in this study.

### Data collection and statistics

Clinical data including sex, body weight, height, age of onset and diagnosis, initial symptoms and laboratory results, response to treatment, and detailed information of *SLCO2A1* variations, were collected. Information about GI tract involvement was collected from the endoscopic, radiologic, and histologic findings. All continuous variables in the blood laboratory results were presented as median with interquartile range (IQR), and all discrete variables were presented as numbers with percentages (%). All z-scores of height and weight were analyzed using 2017 Korean national growth charts. [26] Also, we used the dataset of Genome Aggregation Database (gnomAD), version 2.1, to analyze the population frequencies of each *SLCO2A1* mutation revealed. [27].

### Molecular diagnosis

Blood samples were drawn from all enrolled patients for molecular genetic testing of *SLCO2A1*. Sanger sequencing of *SLCO2A1* was performed for all patients. Genomic DNA was isolated from peripheral blood leukocytes from the study subjects. All 14 coding exons of *SLCO2A1* and their intronic flanking regions were PCR-amplified using specific primer pairs. Amplification was conducted over 30 cycles, and the PCR mixtures were separated on 1.5% agarose gels to confirm the size and purity of the PCR products. Subsequently, DNA sequencing reactions were carried out, and the reaction mixtures were analyzed using an ABI3130xl Genetic Analyzer (Applied Biosystems, CA, USA) and Sequencing Analysis v.5.2 software. To predict the functional impact of novel amino acid changes, we carried out molecular analyses of the patients’ parents and 100 healthy controls, and compared the results with the patients’ results. Additionally, we assessed novel missense alterations using in silico prediction algorithms. The suspected variants were further screened using the ExAC browser, gnomAD, or the 1000 Genome Project dataset.

## Results

### Baseline and endoscopic features of pediatric CEAS

Table 1 shows the clinical baseline information and endoscopic features of pediatric patients with CEAS. Among them, seven were male and four were female. The median age at symptom onset was only 4.0 years (IQR 2.0 ~ 6.0), and the median age at diagnosis was 16.0 years (IQR 11.0 ~ 20.0). The Median z-score of height at diagnosis was -0.44 (IQR -0.90 ~ 0.03), and the median z-score of weight was -0.87 (-1.40 ~ -0.26). A family history of CEAS was identified in two patients, and specifically, two siblings shared the same pathogenic *SLCO2A1* mutations. This family is not consanguineous, and family genetic testing confirmed that each parent carries one allele, respectively. Patients had various diagnoses in the past, with the majority having iron deficiency anemia (IDA). Among previous diagnoses related to the GI tract, EGID was the most prevalent, followed by duodenal ulcer and CMUSE.

**Table 1:** Baseline information and endoscopic findings of the patients

Number of patients	11
Median age, years (IQR)	
Diagnosis	16.0 (11.0 ~ 20.0)
Symptom onset	4.0 (2.5 ~ 6.0)
Z-score at diagnosis, median (IQR)	
Height	-0.44 (-0.90 ~ 0.03)
Weight	-0.87 (-1.40 ~ -0.26)
Gender, No. (%)	
Male	7 (63.6)
Female	4 (36.4)
A family history of CEAS, No. (%)	2 (18.2)
Previous diagnosis, No. (%)	
Iron deficiency anemia	9 (81.9)
Eosinophilic gastrointestinal disease	4 (36.4)
Duodenal ulcer	3 (27.3)
CMUSE	3 (27.3)
Protein losing enteropathy	1 (9.1)
Involved GI site, No. (%)	
Esophagus	0 (0)
Stomach	5 (45.5)
Fundus or body	0 (0)
Prepyloric area	5 (45.5)
Small intestine	11 (100)
Duodenum	7 (63.6)
Jejunum	4 (36.4)
Ileum	7 (63.6)
Terminal ileum	1 (9.1)
Colon	0 (0)
Type of lesion, No. (%)	
Intestinal ulcer	11 (100)
Intestinal stenosis	6 (54.5)
Patients with History of small intestine resection, No. (%)	5 (45.5)
Cause of bowel surgery, No. (%)	
Capsule endoscopy retention	2 (18.2)
Severe intestinal stenosis	2 (18.2)
Intractable intestinal bleeding	1 (9.1)

*IQR* Interquartile range, *No* Number, *GI* Gastrointestinal, *CMUSE* Cryptogenic multifocal ulcerous stenosing enteritis, *CEAS* Chronic enteropathy associated with *SLCO2A1* gene

All patients exhibited involvement of the GI tract, with 100% exhibiting small intestine involvement. Conversely, the esophagus, fundus or body portion of the stomach, and colon were unaffected in any patient (0%). The most affected GI site was the duodenum and ileum (63.6%), followed by prepyloric area (45.5%) and jejunum (36.4%). Terminal ileum involvement was noted in one patient (9.1%). When investigating the types of lesions, ulcerative lesions were observed in all patients (100%), and intestinal stenosis was identified in 6 patients (54.5%). Small intestine resection was performed in 5 patients (45.5%) due to various reasons; Capsule endoscopy (CE) retention (18.2%), severe intestinal stenosis (18.2%), and intractable intestinal bleeding (9.1%).

**Fig. 1:**
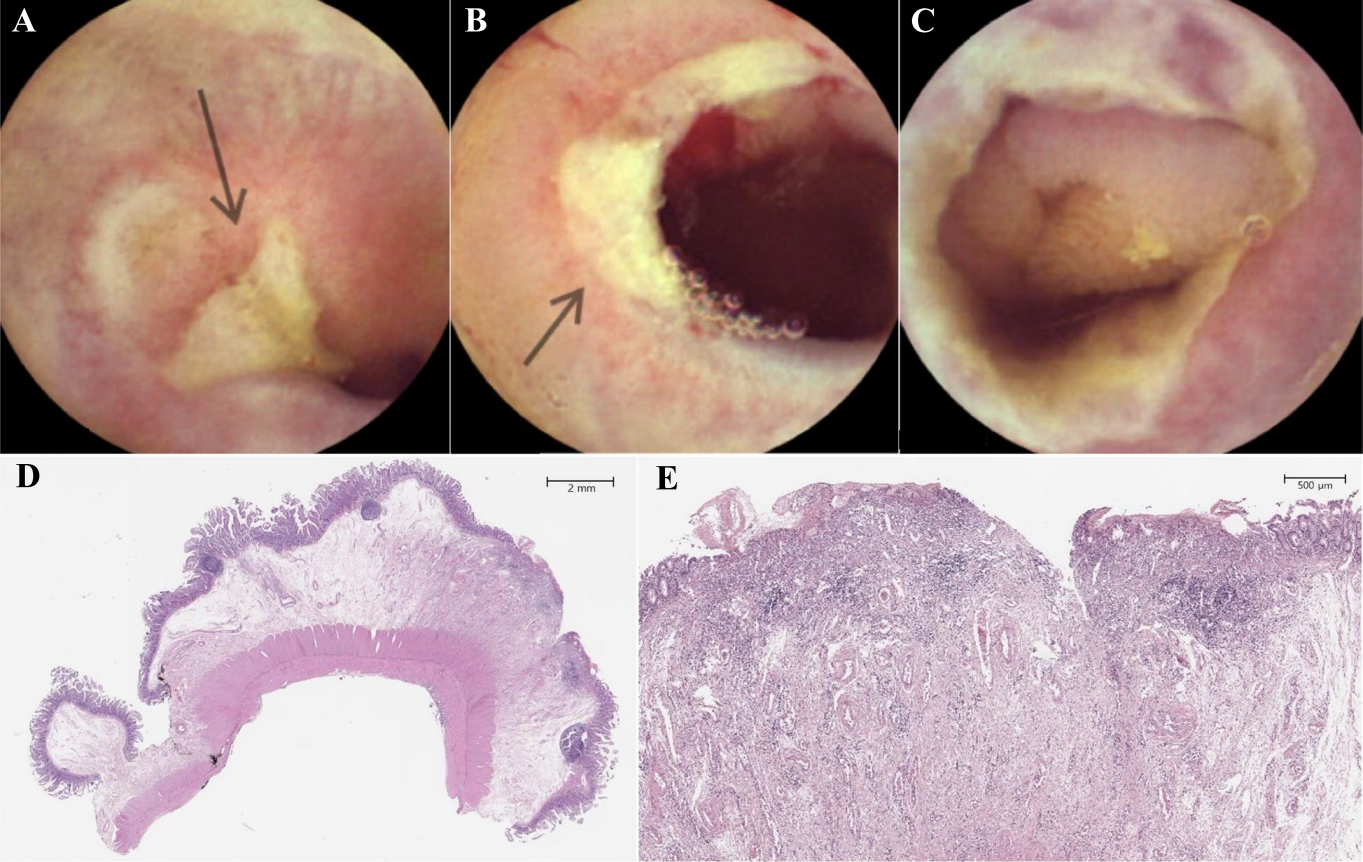
**A** An active ulcer in a capsule endoscopic image. **B** Encircling ulcers with bleeding in a capsule endoscopic image. **C** Encircling ulcers causing a luminal stricture, leading to retention of the capsule endoscopy. **D** A scanned view of an intestinal specimen demonstrating focal ulceration with mucosal and submucosal fibrosis. **E** A magnified view showing focal inflamed granulation tissue, which are indicative of chronic inflammatory changes

**Fig. 2:**
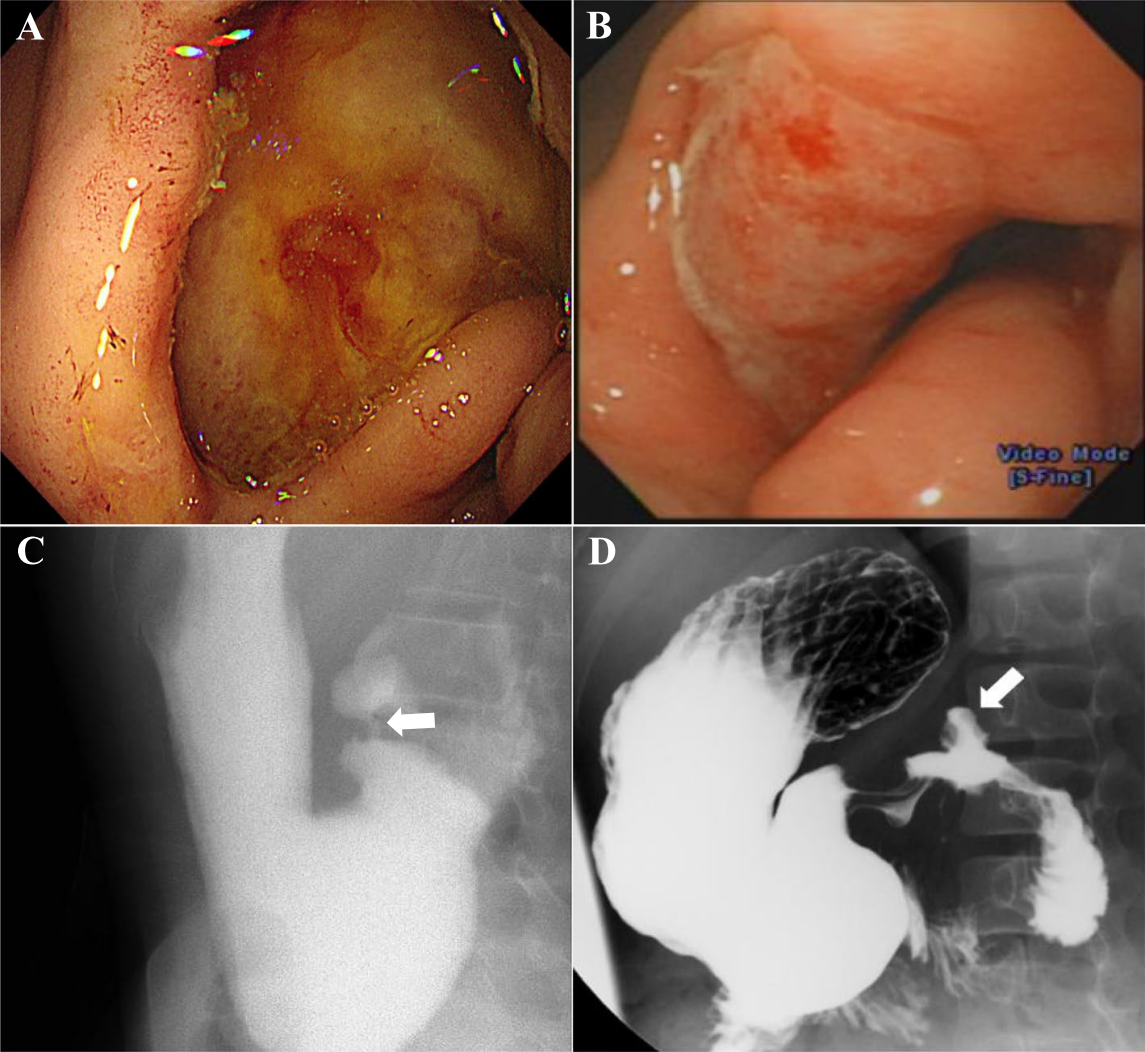
**A** A deep large ulcer on the prepyloric area. **B** A broad large ulcer on the duodenal bulb. **C** An upper gastrointestinal series (UGIS) image showing pyloric stenosis (white arrow). **D** An UGIS Image showing a deformed duodenal bulb with ulceration (white arrow)

Figure 1 shows capsule endoscopic images and histopathological views of multiple ulcers and stricture in the small intestine. Figure 2 shows the endoscopic and upper gastrointestinal series findings of prepyloric or duodenal ulcers with stricture in pediatric CEAS.

### Identified *SLCO2A1* mutations in CEAS

We identified four different *SLCO2A1* mutations, and detailed information on *SLCO2A1* variations is described in Table 2. Overall, 4 patients had homozygous mutations and 7 patients showed heterozygosity. Among the identified *SLCO2A1* mutations, nucleotide change of c.940 + 1G > A (intron 7) was the most common mutation in our patients, followed by c.1807C > T (exon13). These two mutations were known as pathogenic variants according to the American College of Medical Genetics and Genomics and the Association for Molecular Pathology (ACMG/AMP) variant classification guidelines. [28] In our patients, we discovered two novel mutations, c.1329_1344del and c.1350C > G. Both of these mutations were located in exon 10, representing frameshift and missense mutations, respectively. According to the ACMG/AMP variant classification guidelines, the c.1329_1344del mutation can be classified as a pathogenic variant, and the c.1350C > G mutation can be classified as a likely pathogenic variant. [28] Through this study, we revealed a total of 5 different combinations of *SLCO2A1* mutations; c.940G > A homozygote (3 patients), c.1807C > T homozygote (2 patients), c.1807C > T / c.940 + 1G > A (4 patients), c.940 + 1G > A / c.1329_1344del (1 patient), and c.940 + 1G > A / c.1350C > G (1 patient). There was no definite genotype–phenotype correlation among the mutation combinations.

**Table 2:** Detailed information of SLCO2A1 mutations

Chromosome	Genomic position (GRCh37)	Site	Nucleotide change^a^	Predicted effect	Molecular consequence	Mutant allele frequency	Classification	gnomAD allele frequency (allele count/allele number)	
								European (non-Finnish)	East Asian
3	133,654,625	Exon 13	c.1807C > T	p.Arg603*	Nonsense	8/22	P	0.00001788 (2/111,868)	0.0002763 (5/18,096)
3	133,667,736	Intron 7	c.940 + 1G > A	p.Arg288Glyfs*7	Splice site	12/22	P	0.000008790 (1/113,762)	0.0003262 (6/18,394)
3	133,664,068	Exon 10	c.1329_1344del	p.Cys444Argfs*31	Frameshift	1/22	P	0	0
3	133,664,050	Exon 10	c.1350C > G	p.Cys450Trp	Missense	1/22	LP	0	0

^a^The Genbank accession number of SLCO2A1 is NM005630

*GRCh37* Genome Reference Consortium Human build 37, *gnomAD* Genome Aggregation Database, *P* Pathogenic, *LP *Likely pathogenic

Population frequencies of each *SLCO2A1* mutation were further analyzed by GnomAD database. The allele frequency of the c.1807C > T variant in East Asian was 5/18,096, which was much higher than that of European (2/111,868). The allele frequency of the c.940 + 1G > A variant was also much higher in East Asian (6/18,394) than in European (1/113,762). The c.1329_1344del and c.1350C > G variants have not been identified.

### Symptoms and laboratory findings of pediatric CEAS

Table 3 shows clinical symptoms and initial laboratory findings of the patients. Abdominal pain (81.8%) and pale face (72.7%) were the most common symptoms of CEAS, whereas diarrhea (10.0%) and hematochezia (18.2%) was rarely observed. The typical symptoms of PHO, specifically pachydermia, digital clubbing, and periostosis, were observed in 3, 4, and 2 patients, respectively. Notably, when considering only the initial symptom, pale face (72.7%) was predominant, followed by abdominal pain (18.2%).

**Table 3:** Clinical presentations and laboratory findings

Symptoms, No. (%)	
Abdominal pain	9 (81.8)
Pale face	8 (72.7)
Diarrhea	1 (9.1)
Melena	2 (18.2)
Pachydermia	3 (27.3)
Digital clubbing	4 (36.4)
Periostosis	2 (18.2)
Initial blood laboratory results, median (IQR)	
Hemoglobin (g/dl)	9.5 (7.2 ~ 11.1)
Total protein (g/dl)	5.0 (4.6 ~ 5.0)
Albumin (g/dl)	3.1 (3.0 ~ 3.7)
CRP^a^ (mg/dl)	0.2 (0.1 ~ 1.9)
Ferritin (ng/ml)	6.9 (3.0 ~ 11.5)
Iron (μg/dl)	15.0 (11.5 ~ 19.5)
Iron saturation (%)	3.90 (2.70 ~ 5.03)
Initial stool laboratory results, No. (%)	
Occult blood positive^b^	8 (100.0)
Calprotectin elevation (> 250 μg/g)^b^	3 (37.5)

*IQR* Interquartile range, *CRP* C-reactive protein, *No* Number

^a^Initial CRP levels were performed in 9 patients

^b^Initial stool occult blood and calprotectin level tests were performed in 8 patients

In the initial blood laboratory results, the median level of hemoglobin was 9.5 g/dl (IQR 7.2 ~ 11.1). The median levels of total protein and albumin were 5.0 g/dl (IQR 4.6 ~ 5.0) and 3.1 g/dl (IQR 3.0 ~ 3.7), respectively. The median level of C-reactive protein (CRP) was 0.2 (IQR 0.1–1.9). The median levels of ferritin and iron saturation were 6.9 ng/ml (IQR 3.0 ~ 11.5) and 3.90% (IQR 2.70 ~ 5.03), respectively.

Fecal occult blood tests and calprotectin levels were initially assessed in 8 patients. The initial fecal occult blood tests showed positive results in all patients (100%), and elevated fecal calprotectin levels were observed in 3 patients (37.5%).

### Treatment history and clinical course

All patients were treated based on their previous diagnoses, and the clinical course varied widely from case to case. All patients had a history of using anti-acid agents, and among them, only two patients experienced symptom improvement with anti-acid agents. Currently, they do not report any gastrointestinal symptoms without any medications.

Nine patients have received immunomodulatory drugs more than once. They all have been taking iron supplements for years due to chronic IDA. Among them, eight patients were treated with azathioprine (1 ~ 3 mg/kg/day, max dose: 100 mg) for a minimum duration of six months or longer, and only two have continued so far. The remaining six patients discontinued or switched to other immunomodulatory drugs due to a lack of clinical response. Two patients were administered Infliximab (5 mg/kg intravenously, with induction doses given at 0, 2, and 6 weeks, followed by maintenance doses every 8 weeks) over a period of two years, but it did not show significant improvement in the disease. In only one patient, during the infliximab maintenance period, infliximab trough levels and anti-infliximab antibody levels were measured, with levels of < 0.1 μg/mL and high titer, respectively. Considering these lab results and the lack of symptom improvement, we determined that the patient experienced a loss of response to infliximab, leading to discontinuation. Partial enteral nutrition providing 50% of the patient’s resting energy expenditure was attempted in one patient for 5 years, but it proved ineffective. In conclusion, so far, none of the treatments including immunomodulatory drugs and elemental diets has shown significant effect in improving symptoms and laboratory abnormalities in patients with CEAS.

## Discussion

Through this study, we identified 11 pediatric CEAS patients, representing the largest reported number of pediatric CEAS cases to date. We also found with two novel mutations of *SLCO2A1*, and analyzed the detailed characteristics and treatment response of pediatric CEAS. Though CEAS is gradually recognized as one type of monogenic IBD making it essential to understand the clinical manifestations in pediatric populations, there is scarce literature reporting pediatric CEAS. [22, 25].

In our study, the majority of patients exhibited CEAS-related symptoms from an early age. The median age at symptom onset is only 4.0 years, indicating that CEAS typically develops from a very early age. Recent papers have classified CEAS as a subtype of monogenic very early onset inflammatory bowel disease (VEO-IBD), defined as an IBD that develops in children under the age of 6. [22, 24] However, the evidence supporting the early onset of CEAS is limited to date. Through this study, we present significant evidence indicating that CEAS can manifest at a very early age, and be classified as monogenic VEO-IBD.

All patients in our study exhibited ulcers in the GI tract, and intestinal strictures were also frequently observed. The lesions were observed between the prepyloric area and the terminal ileum, with no involvement of the esophagus and colon, which is similar with previous adult reports. [8, 9] While the involvement of the terminal ileum in CEAS remains controversial, we found one patient with lesions in the terminal ileum, suggesting that CEAS can affect the entire small intestine. [9, 14, 20] Furthermore, a significant number of patients had a history of small intestine resection, with two cases attributed to the retention of CE. Previously there were some reports about CE retention in CMUSE or IBD patients. [7, 29, 30] Given the high incidence of intestinal stricture, performing CE in suspected CEAS patients should require caution. We recommend initiating the assessment with a patency capsule as a safety measure.

Our study revealed that abdominal pain and pale face were the most common symptoms of pediatric CEAS. We also identified patients with clinical features of PHO, which share the same pathogenic gene with CEAS. There have been reports about GI symptoms of PHO in a family, and patients of CEAS with PHO have also been reported. [20, 31–34] In addition, we identified IDA and hypoalbuminemia, without marked systemic inflammation, as major initial characteristics of CEAS. Because CEAS predominantly affects the mucosal and submucosal area, it is thought to manifest as shallow intestinal ulcers and does not result in elevated inflammatory markers. And, unlike Crohn’s disease, the elevation of fecal calprotectin levels was not typical in CEAS. Though melena was uncommon, the initial fecal occult blood tests all showed positive findings. These results suggest that CEAS differs from other major IBDs, and induces persistent microscopic GI bleeding, associated with protein and iron depletion. Furthermore, they indicate the necessity of conducting *SLCO2A1* sequencing for patients with chronic iron deficiency anemia from very early age and normal CRP levels, along with confirmed small intestinal lesions.

Most patients in our study continue to experience intractable symptoms despite undergoing various immunomodulatory medications. For this reason, it is believed that patients showed relatively short height and low weight for age at the time of diagnosis. The treatment of CEAS has not been established yet, and although one study reported the efficacy of azathioprine, it did not demonstrate effectiveness in the majority of our patients. [13] Due to the refractoriness of CEAS exacerbating growth delay in children, continuous research on treatment of CEAS is essential in the future.

We found two novel pathogenic *SLCO2A1* mutations in CEAS, c.1329_1344del and c.1350C > G. The most common pathogenic variant in our study was c.940 + 1G > A followed by c.1807C > T, which were also most frequently found in the previous report. [8] Furthermore, according to gnomAD dataset, incidence of both mutations was much higher in East Asian than in European. [27] The cause of ethnical differences in the incidence of CEAS is not yet clear, but this is likely associated with most CEAS patients being reported in Asian populations. [9, 14, 17].

Pathophysiology of CEAS is still unclear. *SLCO2A1* is known to encode prostaglandin transporter (PGT), which mediates the uptake, release, and clearance of prostaglandin (PG) from cells in multiple organs, and is also expressed in the mucosa of the GI tract. [10, 35–38] A previous study showed markedly decreased PGT expression in vascular endothelial cells in the intestine of CEAS compared to that in the control group. [39] In addition, PG is known to be associated with both pro-inflammatory and anti-inflammatory effects in the GI tract, and it also contributes to the protection and healing of intestinal mucosa. [38, 40] One study noted that loss of *SLCO2A1* increases the concentration of PGE_2_ in the intestinal tissue, resulting in activation of macrophages. [41] These studies indicate that intracellular prostaglandin dysregulation of GI tract might be associated with CEAS, and further research on the pathophysiology of CEAS will be necessary.

This study had some limitations. Firstly, the number of CEAS patients in our study was small. However, given the rarity of the disease, it is noteworthy that we exclusively collected data from pediatric patients, resulting in the largest study of pediatric CEAS. Secondly, full endoscopic examinations for evaluating the entire small intestine were not performed in all patients; hence, there is a possibility that other lesions of the small intestine may not be fully identified. In this respect, a few patients aimed to indirectly assess the small intestine through imaging tests such as computed tomography or magnetic resonance enterography. Thirdly, due to the short duration since diagnosis of CEAS, there were limitations in including long term data of pediatric CEAS and providing detailed treatment efficacy of various immunomodulatory medications. Furthermore, the lack of data on infliximab treatment escalation and drug levels of medications such as azathioprine is also considered an important limitation. Therefore, a study on the follow-up of CEAS patients in the future is necessary to assess treatment response and long term prognosis.

## Conclusion

Our study analyzed the clinical characteristics of pediatric CEAS. We revealed that CEAS commonly manifests from a very young age with growth delay and can be classified as a subtype of monogenic VEO-IBD. CEAS is characterized by multiple ulcers and strictures from the prepyloric area to the terminal ileum, while sparing the esophagus and colon. *SLCO2A1* mutations should be evaluated in patients with chronic IDA from a very early age and small intestine ulcers, particularly in cases where CRP levels are not elevated. To date CEAS is regarded as an intractable disease leading to growth delay in childhood; thus, further studies are needed to investigate the pathophysiology and treatment of pediatric CEAS.

## Data Availability

All datasets generated from this study are included in this article.

## Abbreviations

CEAS: Chronic enteropathy associated with *SLCO2A1* gene
IDA: Iron deficiency anemia
IBD: Inflammatory bowel disease
CNSU: Chronic nonspecific multiple ulcers of the small intestine
CMUSE: Cryptogenic multifocal ulcerous stenosing enteritis
PHO: Primary hypertrophic osteoarthropathy
GI: Gastrointestinal
EGID: Eosinophilic gastrointestinal disease
VEO-IBD: Very early onset inflammatory bowel disease
IRB: International review board
IQR: Interquartile range
gnomAD: Genome aggregation database
PGT: Prostaglandin transporter
PG: Prostaglandin

## References

[1] Doecke JD, Simms LA, Zhao ZZ, Huang N, Hanigan K, Krishnaprasad K (2013). Genetic susceptibility in IBD: overlap between ulcerative colitis and Crohn’s disease. Inflamm Bowel Dis.

[2] de Ridder L, Weersma RK, Dijkstra G, van der Steege G, Benninga MA, Nolte IM (2007). Genetic susceptibility has a more important role in pediatric-onset Crohn’s disease than in adult-onset Crohn’s disease. Inflamm Bowel Dis.

[3] Jakobsen C, Cleynen I, Andersen P, Vermeire S, Munkholm P, Paerregaard A (2014). Genetic susceptibility and genotype–phenotype association in 588 Danish children with inflammatory bowel disease. J Crohns Colitis.

[4] Sýkora J, Pomahačová R, Kreslová M, Cvalínová D, Štych P, Schwarz J (2018). Current global trends in the incidence of pediatric-onset inflammatory bowel disease. World J Gastroenterol.

[5] Okabe H, Sakimura M (1968). Nonspecific multiple ulcer of the small intestine. Stomach and Intestine.

[6] Matsumoto T, Iida M, Matsui T, Yao T (2007). Chronic nonspecific multiple ulcers of the small intestine: a proposal of the entity from Japanese gastroenterologists to Western enteroscopists. Gastrointest Endosc.

[7] Tao EW, Zou TH, Wang YF, Tang JT, Chen YX, Gao QY (2019). Case report of cryptogenic multifocal ulcerous stenosing enteritis (CMUSE): a rare disease may contribute to endoscopy-capsule retention in the small intestine. BMC Gastroenterol.

[8] Hosoe N, Ohmiya N, Hirai F, Umeno J, Esaki M, Yamagami H (2017). Chronic Enteropathy Associated with SLCO2A1 gene [CEAS]—characterisation of an enteric disorder to be considered in the differential diagnosis of Crohn’s disease. J Crohns Colitis.

[9] Umeno J, Esaki M, Hirano A, Fuyuno Y, Ohmiya N, Yasukawa S (2018). Clinical features of chronic enteropathy associated with SLCO2A1 gene: a new entity clinically distinct from Crohn’s disease. J Gastroenterol.

[10] Umeno J, Hisamatsu T, Esaki M, Hirano A, Kubokura N, Asano K (2015). A hereditary enteropathy caused by mutations in the SLCO2A1 gene, encoding a prostaglandin transporter. PLoS Genet.

[11] Niizeki H, Shiohama A, Sasaki T, Seki A, Kabashima K, Otsuka A (2014). The complete type of pachydermoperiostosis: a novel nonsense mutation p. E141* of the SLCO2A1 gene. J Dermatol Sci.

[12] Zhang Z, Xia W, He J, Zhang Z, Ke Y, Yue H (2012). Exome sequencing identifies SLCO2A1 mutations as a cause of primary hypertrophic osteoarthropathy. Am J Hum Genet.

[13] Eda K, Mizuochi T, Takaki Y, Ushijima K, Umeno J, Yamashita Y (2018). Successful azathioprine treatment in an adolescent with chronic enteropathy associated with SLCO2A1 gene: a case report. Medicine..

[14] Huang H, Wang X, Ou D, Liu X, Wu B, Zhou B, et al. Four variants of SLCO2A1 Identified in Three Chinese patients with chronic enteropathy associated with the SLCO2A1 Gene. Dig Dis Sci. 2020;66:1–10. 10.1007/s10620-020-06629-0 33000396

[15] Sun X, Hosoe N, Miyanaga R, Kimura K, Mizuno S, Takabayashi K (2018). A male Korean who was diagnosed with chronic enteropathy associated with SLCO2A1 (CEAS): case report with literature review. BMJ open Gastroenterol.

[16] Hu P, He H, Dai N, Zhang S, Deng L (2019). Chronic enteropathy associated with SLCO2A1 gene: A case report and literature review. Clin Res Hepatol Gastroenterol.

[17] Jimbo K, Okuno T, Ohgaki R, Nishikubo K, Kitamura Y, Sakurai Y (2020). A novel mutation in the SLCO2A1 gene, encoding a prostaglandin transporter, induces chronic enteropathy. PLoS One.

[18] Sun K, He Q, Zhu L, Abula G, Zhao J, Chen X (2021). Multiple small intestinal ulcers with SLCO2A1 and PLA2G4A mutation in a Chinese patient. Dig Liver Dis.

[19] Yanai S, Yamaguchi S, Nakamura S, Kawasaki K, Toya Y, Yamada N (2019). Distinction between chronic enteropathy associated with the SLCO2A1 gene and Crohn’s disease. Gut Liver.

[20] Hong HS, Baek J, Park JC, Lee H-S, Park D, Yoon A-R (2022). Clinical and Genetic characteristics of korean patients diagnosed with chronic enteropathy associated with SLCO2A1 gene: a KASID multicenter study. Gut Liver.

[21] Hamon A, Cazals-Hatem D, Stefanescu C, Uzzan M, Treton X, Sauvanet A (2023). Crohn-like disease affecting small bowel due to monogenic SLCO2A1 mutations: first cases of chronic enteropathy associated with SLCO2A1 Gene [CEAS] in France. J Crohns Colitis.

[22] Collen LV, Kim DY, Field M, Okoroafor I, Saccocia G, Whitcomb SD, et al. Clinical phenotypes and outcomes in monogenic versus non-monogenic very early onset inflammatory bowel disease. J Crohns Colitis. 2022;16(9):1380–96. 10.1093/ecco-jcc/jjac045 PMC9455789 35366317

[23] Kelsen JR, Sullivan KE, Rabizadeh S, Singh N, Snapper S, Elkadri A (2020). North American Society for Pediatric Gastroenterology, Hepatology, and Nutrition position paper on the evaluation and management for patients with very early-onset inflammatory bowel disease. J pediatr Gastroenterol Nutr.

[24] Ouahed J, Spencer E, Kotlarz D, Shouval DS, Kowalik M, Peng K (2020). Very early onset inflammatory bowel disease: a clinical approach with a focus on the role of genetics and underlying immune deficiencies. Inflamm Bowel Dis.

[25] Uchida K, Nakajima A, Ushijima K, Ida S, Seki Y, Kakuta F (2017). Pediatric-onset chronic nonspecific multiple ulcers of small intestine: a nationwide survey and genetic study in Japan. J Pediatr Gastroenterol Nutr.

[26] Kim JH, Yun S, Hwang SS, Shim JO, Chae HW, Lee YJ (2018). The 2017 Korean National growth charts for children and adolescents: development, improvement, and prospects. Korean. J Pediatr.

[27] Karczewski KJ, Francioli LC, Tiao G, Cummings BB, Alföldi J, Wang Q (2020). The mutational constraint spectrum quantified from variation in 141,456 humans. Nature.

[28] Richards S, Aziz N, Bale S, Bick D, Das S, Gastier-Foster J (2015). Standards and guidelines for the interpretation of sequence variants: a joint consensus recommendation of the American college of medical genetics and genomics and the association for molecular pathology. Genet Med.

[29] Li F, Gurudu SR, De Petris G, Sharma VK, Shiff AD, Heigh RI (2008). Retention of the capsule endoscope: a single-center experience of 1000 capsule endoscopy procedures. Gastrointest Endosc.

[30] Pasha SF, Pennazio M, Rondonotti E, Wolf D, Buras MR, Albert JG (2020). Capsule retention in Crohn’s disease: a meta-analysis. Inflamm Bowel Dis.

[31] Sethuraman G, Malhotra A, Khaitan B, Sharma V, Kumar R, Makharia G (2006). Familial pachydermoperiostosis in association with protein-losing enteropathy. Clin Exp Dermatol.

[32] Kim WH, Go YW, Lee CR, Kang E, Kwon KW, Kim HG (1999). Crohn’s Disease As sociated with Pachydermoperiostosis. Korean J Gastroenterol.

[33] Compton RF, Sandborn WJ, Yang H, Lindor NM, Tremaine W, Davis M (1997). A new syndrome of Crohn’s disease and pachydermoperiostosis in a family. Gastroenterology.

[34] Tsuzuki Y, Aoyagi R, Miyaguchi K, Ashitani K, Ohgo H, Yamaoka M (2020). Chronic enteropathy associated with SLCO2A1 with pachydermoperiostosis. Intern Med.

[35] Kanai N, Lu R, Satriano JA, Bao Y, Wolkoff AW, Schuster VL (1995). Identification and characterization of a prostaglandin transporter. Science.

[36] Nomura T, Lu R, Pucci ML, Schuster VL (2004). The two-step model of prostaglandin signal termination: in vitro reconstitution with the prostaglandin transporter and prostaglandin 15 dehydrogenase. Mol Pharmacol.

[37] Mandery K, Bujok K, Schmidt I, Wex T, Treiber G, Malfertheiner P (2010). Influence of cyclooxygenase inhibitors on the function of the prostaglandin transporter organic anion-transporting polypeptide 2A1 expressed in human gastroduodenal mucosa. J Pharmacol Exp Ther.

[38] Dey I, Lejeune M, Chadee K (2006). Prostaglandin E2 receptor distribution and function in the gastrointestinal tract. Br J Pharmacol.

[39] Yamaguchi S, Yanai S, Nakamura S, Kawasaki K, Eizuka M, Uesugi N (2018). Immunohistochemical differentiation between chronic enteropathy associated with SLCO2A1 gene and other inflammatory bowel diseases. Intest Res.

[40] Takeuchi K, Amagase K (2018). Roles of cyclooxygenase, prostaglandin E2 and EP receptors in mucosal protection and ulcer healing in the gastrointestinal tract. Curr Pharm Des.

[41] Nakata R, Nakamura Y, Hosomi S, Okuda H, Nishida Y, Sugita N (2020). Slco2a1 deficiency exacerbates experimental colitis via inflammasome activation in macrophages: a possible mechanism of chronic enteropathy associated with SLCO2A1 gene. Sci Rep.

